# School Belonging and STEM Career Interest in Chinese Adolescents: The Mediating Role of Science Identity

**DOI:** 10.3390/bs15101365

**Published:** 2025-10-07

**Authors:** Yuling Li, Yan Kong

**Affiliations:** School of Humanities and Social Sciences, University of Science and Technology of China, Hefei 230026, China; lyl0721@mail.ustc.edu.cn

**Keywords:** school belonging, STEM career interest, science identity, Chinese student, mediating effect

## Abstract

Adolescents’ sustained engagement in STEM fields is critical for cultivating future scientific talent. While school belonging—a key form of emotional support perceived by students within the school environment—has been widely studied, its specific influence on STEM career interest, particularly within non-Western educational systems, remains insufficiently understood. Drawing on Social Cognitive Career Theory (SCCT), this study examines how school belonging, as a contextual affordance, shapes STEM career interest among Chinese high school students, and whether science identity, as a person input, mediates this relationship. Utilizing data from 451 students in a science-focused Chinese high school, multiple regression analyses demonstrated that school belonging significantly predicts higher STEM career interest. Science identity partially mediated this relationship, with science interest emerging as the strongest mediating component, followed by competence/performance beliefs; external recognition had a comparatively weaker effect. These findings suggest that fostering school belonging in science-oriented environments may support adolescents’ interest in STEM careers, both directly and indirectly through the development of science identity. From a cultural perspective, the study further sheds light on the mechanisms underlying students’ interest in STEM careers, and highlights the potential of inclusive environments that support the development of students’ sense of belonging and identity in promoting their long-term engagement in STEM fields.

## 1. Introduction

The cultivation of STEM (Science, Technology, Engineering, and Mathematics) talent is widely recognized as a cornerstone of national competitiveness and global innovation. Forming an interest in STEM careers during adolescence is particularly consequential, as it predicts later choices of advanced coursework, degree attainment, and eventual entry into the STEM workforce (Kier et al., 2014; Maltese & Tai, 2010; Nitzan-Tamar & Kohen, 2022). Nevertheless, international evidence suggests a steady decline in young people’s enthusiasm for STEM fields, a pattern often described as a “leaky pipeline”, whereby aspirations diminish as students advance through schooling (DeWitt et al., 2014; Z. Jiang et al., 2024).

In China, the challenge of sustaining adolescents’ STEM engagement is particularly pressing. Although mainland China did not fully participate in PISA 2022 due to COVID-19 disruptions, earlier cycles had already revealed low science career interest—for instance, only 16.7% of Chinese students in PISA 2015 intended to pursue science by age 30, compared to 37.9% in the United States (OECD, 2016). National evidence is consistent: fewer than one in five eighth graders expressed interest in STEM careers (MOE, 2021), and a 2023 baseline survey likewise reported generally low career expectations (China Association for Science and Technology & UNICEF, 2023). Declining high school enrollment in core science subjects such as physics, chemistry, and biology further illustrates this concern (Chinese Journal of Science, 2023; Ni, 2023). In response, China has recently launched a series of coordinated reforms to strengthen science education and foster sustained interest in STEM. These efforts include talent development initiatives, such as the National Basic Subject Talent Training Plan (1.0 and 2.0), which aim to identify and nurture students’ scientific potential from an early stage by linking academic growth with clear future pathways. They also encompass student-centered environmental building and curriculum reforms—such as the 2022 Compulsory Education Science Curriculum Standards and the Opinions on Strengthening Science Education in Primary and Secondary Schools in the New Era (2023)—designed to promote a sense of connection, recognition, and active participation within the school community. However, empirical evidence remains scarce within the context of China’s rapid reforms and distinctive cultural conditions.

Considering the school’s central role in adolescent development and the notion that belonging represents a basic psychological need, school belonging has consistently been linked to students’ motivation, long-term interests, and academic achievement, with those who feel a strong sense of belonging more likely to engage in learning, persist through challenges, and maintain optimism about their future (K. Allen et al., 2018; Finn, 1989; Goodenow, 1993a; Pittman & Richmond, 2007). Since 2012, the Programme for International Student Assessment (PISA) has recognized school belonging as a key indicator of student well-being and engagement (OECD, 2013, 2016, 2019, 2023). Complementing this, analyses of TIMSS data show that students’ sense of belonging significantly predicts enjoyment and confidence in science and mathematics across multiple countries, despite notable national differences (Smith et al., 2020, 2021, 2022). Nevertheless, the specific mechanisms through which school belonging shapes STEM career interest remain insufficiently understood, particularly in non-Western contexts.

Furthermore, research on students’ choices, persistence, and withdrawal from STEM has increasingly emphasized the importance of science identity—the extent to which students perceive themselves, and are recognized by others, as competent participants in scientific practices (Carlone & Johnson, 2007). Science identity has been shown to predict long-term engagement with science even after accounting for prior achievement (Vincent-Ruz & Schunn, 2018), and contextual factors such as gender and school belonging further shape its development (Seyranian et al., 2018). Taken together, these insights suggest that school belonging may foster the development of science identity, which in turn channels students’ motivation toward STEM career interest—a pathway that remains underexamined, particularly in the Chinese context.

While research has emphasized the importance of school belonging and scientific identity, few studies have examined how they interact to influence adolescents’ STEM career interest within the Chinese context. The present study addresses this gap by proposing a conceptual framework that integrates both dimensions to investigate their joint influence on STEM career interest. The study is situated in a STEM-focused model high school in eastern China, a provincial exemplar that combines rigorous STEM curriculum with rich opportunities for informal learning. Unlike most Chinese high schools, which offer limited extracurricular science opportunities, this school operates within a resource-rich STEM ecosystem characterized by elite resources, structured competitions, and mentorship from doctoral-level scientists. Such a distinctive context underscores the importance of situating psychosocial processes within specific educational environments and provides an ideal case for examining how school belonging and science identity jointly influence adolescents’ STEM career interest.

### 1.1. School Belonging and Students’ STEM Career Interest

School belonging has been conceptualized using a variety of overlapping terms, including school bonding, attachment, engagement, connectedness, and community (K. Allen et al., 2018). Among these perspectives, the most widely accepted definition refers to the extent to which students feel personally accepted, respected, included, and supported within the school social environment (Goodenow, 1993a, 1993b). This definition forms the basis for most contemporary empirical and theoretical work on school belonging (K. Allen et al., 2018).

Extensive empirical research has documented the positive outcomes associated with school belonging. Students who feel accepted and supported at school are more likely to participate actively in learning, persist through academic challenges, and maintain optimism about their future trajectories (Finn, 1989; Pittman & Richmond, 2007). Belonging is also positively related to socio-emotional development, motivation, mental health, and academic self-efficacy (Anderman, 2002; Battistich et al., 1997; Hagborg, 1998; Slaten et al., 2016). However, these effects are not automatic; they operate through specific psychological mechanisms and differ across contexts.

Belongingness Motivation Theory posits that the need to belong is a basic human motive that shapes cognition, emotion, and behavior (Baumeister & Leary, 1995). When students feel included, they are more likely to engage meaningfully in learning—especially in STEM domains where feelings of exclusion are prevalent (Faircloth & Hamm, 2005; Hoffman et al., 2021a). Complementarily, Social Cognitive Career Theory (SCCT) emphasizes that self-efficacy and outcome expectations are key predictors of career interest (Lent et al., 1994), and school belonging supports these constructs by enhancing students’ confidence and belief that their efforts will be rewarded (Gillen-O’Neel & Fuligni, 2013; Hoffman et al., 2021b). Cross-national research illustrates how belonging operates differently across settings: it enhances resilience for marginalized students in STEM (Fong et al., 2024), sustains motivation and well-being in Australia (K. A. Allen et al., 2016, 2017), and buffers systemic disadvantages in Nordic contexts (Hansen et al., 2024). Recent scholarship further underscores the contextual complexity of belonging. Kuttner (2023) argues that belonging should be conceptualized as agentic, intersectional, systemic, political, place-based, and a right, rather than a simple psychological state. Moreover, for underrepresented students, belonging may be conditional upon conforming to dominant norms or suppressing aspects of their identity (Chiu et al., 2025). Taken together, these perspectives highlight that belonging is not a universal feeling but a contextual affordance embedded in relational and institutional structures that can either empower or constrain students’ long-term motivation.

In China, school belonging is situated within a collectivist framework in which schools are often regarded as moral and social extensions of the family. This orientation is reinforced by the central role of the class collective, where students’ sense of belonging is cultivated through shared routines, collective honor, and strong identification with the homeroom teacher (Ha et al., 2019; Xie & Yang, 2012). At the same time, the examination-oriented culture—especially the dominance of high-stakes testing—links belonging closely to academic performance (Liu, 2017), so that students often feel valued and included to the extent that they contribute to collective success in exams. Teachers, as both knowledge authorities and moral exemplars, play a pivotal role in shaping belonging by combining academic guidance with explicit value education, a tradition rooted in China’s long-standing emphasis on moral cultivation through schooling (Zeng, 2024). Furthermore, extracurricular structures such as science Olympiads, innovation competitions, and mentorship programs function as institutionalized pathways through which recognition and belonging are affirmed (Tschisgale et al., 2024). A recent meta-analysis confirmed a consistent moderate correlation between belonging and academic achievement (Qiu et al., 2024), and national studies further show that students who feel accepted and valued are more likely to internalize school norms, engage in self-regulated learning, and align their goals with collective success (H. Jiang, 2023; Lu, 2024; Pan & Chang, 2024; Xu & Lastrapes, 2022). These dynamics extend directly to STEM: supportive climates characterized by relational trust, extracurricular opportunities, and performance-based recognition significantly enhance students’ motivation and persistence in science learning (Chuan, 2024; Zhang et al., 2021). Yet existing research primarily reports correlations, offering limited insight into how these culturally specific mechanisms of belonging are transformed into the kinds of learning experiences that shape long-term career interest.

SCCT provides a useful framework for clarifying this transformation. Belonging can be understood as a form of contextual support that generates the learning experiences central to SCCT. Specifically, supportive school climates (a) provide mastery opportunities for repeated success in science tasks, (b) enable vicarious learning through access to peers and mentors as role models, (c) strengthen the impact of social persuasion via credible feedback and encouragement, and (d) improve affective states by reducing anxiety and fostering persistence. Empirical evidence shows that these mechanisms are powerful: McMahon et al. (2008) found that, among multiple classroom and school factors, school belonging emerged as the strongest predictor of students’ academic self-efficacy, highlighting its role as a key contextual support. More recent studies confirm a causal pathway from belonging to self-efficacy (Slaten et al., 2025) and demonstrate similar patterns in Chinese settings such as distance learning contexts.

Through these learning experiences, belonging enhances self-efficacy and informs outcome expectations—students come to believe not only that they can succeed in science but also that their efforts will yield valued academic and career opportunities (H. Jiang et al., 2024). Together, these beliefs are proximal drivers of STEM career interest in the SCCT framework (Chan, 2022; Lent et al., 2016). In short, belonging systematically engineers efficacy-building experiences and channels students toward sustained STEM aspirations.

Building on these insights, it is important to recognize that the influence of school belonging on STEM career interest is rarely direct alone. Rather, belonging often shapes students’ motivation through the development of more proximal constructs that anchor self-perceptions and long-term engagement. Among these, science identity—the extent to which students perceive themselves, and are recognized by others, as “science people”—has emerged as a particularly salient mediator. By integrating competence beliefs, interest, and recognition, science identity provides a developmental bridge between the socio-emotional affordances of school environments and adolescents’ aspirations for future careers. The next section elaborates on the structure and sociocultural formation of science identity and its relevance for understanding students’ STEM trajectories in the Chinese context.

### 1.2. Science Identity: Structure and Sociocultural Formation

The concept of science identity has evolved significantly over the past two decades. Initially defined as an individual’s self-recognition of their ability to perform scientific tasks and pursue a career in science (Brickhouse et al., 2000), science identity is now broadly recognized as socially constructed, shaped through both internal evaluations and external recognition (Z. Jiang & Wei, 2025). For instance, Creighton (2007) emphasized the co-construction of identity through self-society interaction, proposing a double-spiral model that integrates personal ability and social practice. Empirical research has also highlighted its developmental salience during adolescence: Vincent-Ruz and Schunn (2018) demonstrated that science identity in middle school significantly predicted subsequent enrollment in science electives and extracurriculars, even after controlling for GPA, self-efficacy, and parental background. Importantly, identity proved to be a stronger predictor of long-term interest than achievement scores, underscoring its value as a motivational driver during this formative stage.

Building on Gee’s identity framework, Carlone and Johnson (2007) advanced a three-dimensional model of science identity, consisting of performance (engagement in scientific practices), competence (mastery of scientific knowledge), and recognition (being seen and acknowledged as a “science person” by oneself and others). This model has been foundational in capturing the interdependence of behaviors and social validation. Later, Hazari et al. (2010) proposed an extended four-dimensional framework, adding interest as a key motivational driver, thereby integrating science identity more explicitly with Social Cognitive Career Theory (SCCT). Their model proved especially relevant in capturing how long-term career commitment is influenced by both internalized interest and social affirmation. (Chen & Wei, 2022; Hazari et al., 2010) Despite this conceptual lineage, both the three-dimensional and four-dimensional models exhibit substantial overlap among dimensions, which hinders the exploration of the unique effects of each component and the comparison of their predictive power across contexts. For example, competence beliefs may be more closely associated with academic persistence, while recognition may better predict long-term STEM engagement among marginalized groups (Vincent-Ruz & Schunn, 2018; Aschbacher et al., 2010).

To address this, Guo et al. (2022) proposed a simplified 3D structure, reorganizing the dimensions into (1) competence/performance beliefs, (2) science interest, and (3) external recognition. This integrated structure allows for empirical parsimony without losing theoretical richness, and has been validated in adolescent and early college student populations in East Asia. In line with this precedent, the present study adopts Guo’s three-dimensional model to maximize the distinction among components while ensuring contextual adaptability to Chinese student populations. It is worth noting, however, that most operationalizations of external recognition—including the present study—primarily emphasize proximal sources such as teachers, peers, and parents. Broader cultural and institutional forms of recognition (e.g., national policies, media portrayals, and public discourse) are also highly relevant but remain underexamined, representing an important direction for future research.

Within the SCCT framework, science identity serves as the proximal psychological mechanism through which contextual affordances such as school belonging shape career interest. Belonging-rich environments generate learning experiences in the form of mastery opportunities, vicarious exposure to role models, credible social persuasion, and supportive affective states. These experiences enhance self-efficacy and cultivate positive outcome expectations—beliefs about the rewards of engaging with science—which are then internalized in the three dimensions of science identity (Hazari et al., 2010; Guo et al., 2022). In this way, identity functions as a developmental bridge that channels everyday socio-emotional experiences into durable STEM aspirations, thereby extending SCCT’s explanatory power in adolescent contexts. To analyze this mediational pathway with clarity, however, it is necessary to specify which type of identity is most relevant, since students’ trajectories can diverge across STEM subdomains.

Although science identity and STEM identity are sometimes used interchangeably, conflating the two obscures important disciplinary distinctions. Empirical studies show that students often follow heterogeneous identity trajectories: some are drawn more toward science and experimentation, others toward engineering design or technology applications (Grimalt-Álvaro et al., 2022). Moote et al. (2020) found that science capital better predicts aspirations in science and engineering than in mathematics or technology. These distinctions are especially important in the Chinese context, where curriculum and high-stakes exams place far greater emphasis on traditional sciences (physics, chemistry, biology) than on engineering or applied technology (Z. Jiang et al., 2024). For this reason, the present study focuses specifically on science identity, which is developmentally salient for adolescents and directly shaped by China’s exam-oriented, science-dominant educational culture.

In the Chinese context, the three dimensions of science identity are shaped in distinctive ways by the exam-oriented and collectivist education system. Competence/performance beliefs are strongly reinforced through standardized test scores, Olympiad achievements, and selective competitions, which serve as socially validated markers of ability and often determine access to elite educational pathways. Science interest, though increasingly emphasized in reforms such as the 2022 Compulsory Education Science Curriculum Standards and the STEM Education 2035 Action Plan, is frequently constrained by high-stakes examinations that limit exploratory opportunities and channel curiosity toward performance outcomes. External recognition tends to be highly institutionalized: rather than stemming from spontaneous peer or teacher validation, it is often embedded in awards, rankings, and collective honors that normalize recognition within structured hierarchies. In elite schools, where such recognition is pervasive, its motivational salience may be diluted compared with the stronger influence of intrinsic interest or competence beliefs. Taken together, these dynamics demonstrate how China’s educational culture not only defines the content of science identity but also calibrates the relative weight of its components in shaping students’ long-term engagement with science.

Recent Chinese scholarship has reconceptualized science identity from a culturally grounded perspective, viewing it not just as a psychological construct but as a normative marker of civic and moral development. Huang and Pei (2024) proposed a three-phase model encompassing value internalization, social activation, and task-based validation. Deng and Tan (2024), analyzing data from over 4000 primary and secondary students, found that science identity was the strongest predictor of science career aspiration, surpassing even academic self-concept. These findings also highlight moderating influences such as gender, the urban–rural divide, and differential access to extracurricular resources, all of which reflect China’s broader sociocultural and structural landscape. Taken together, they suggest that science identity in China is not merely a psychological construct but is deeply shaped by the institutional logics and cultural values of its educational system.

In sum, science identity emerges as a dynamic construct shaped by both individual experiences and sociocultural forces. China’s exam-oriented and science-dominant education system provides a contextually grounded lens for understanding how students connect school experiences with long-term STEM aspirations. By positioning science identity as the key mediator between school belonging and career interest, this study establishes a culturally relevant framework for analyzing adolescents’ engagement with science and addressing motivational challenges in STEM education.

### 1.3. Analytical Framework and Research Hypotheses

Building on the preceding review, the present study examines how school belonging shapes Chinese adolescents’ STEM career interest, with science identity serving as a key mediating mechanism. Extending the SCCT perspective introduced above, we use it as the guiding framework to link contextual affordances, person inputs, and career outcomes in a culturally grounded model.

SCCT, rooted in Bandura’s (1986) social cognitive theory, explains how career interests emerge through self-efficacy beliefs and outcome expectations, which are shaped by learning experiences, person inputs, and contextual affordances (Lent et al., 1994). This framework provides a theoretical basis for analyzing how supportive school contexts translate into durable motivational trajectories across adolescence.

Within SCCT, school belonging can be understood as a contextual affordance that generates formative learning experiences. Supportive school climates offer students mastery opportunities, credible encouragement, vicarious role models, and reduced anxiety—conditions that strengthen self-efficacy (belief in one’s capability to succeed in science) and foster positive outcome expectations (belief that engaging in science will lead to valued rewards). These psychological resources, in turn, are internalized within students’ science identity, which channels current experiences into long-term STEM aspirations. In this way, belonging operates not simply as an emotional state but as an enabling context that shapes the motivational pathways emphasized in SCCT (Slaten et al., 2025; H. Jiang et al., 2024; Chan, 2022).

In operational terms, the SCCT framework is mapped onto this study as follows: (1) school belonging represents contextual affordances, (2) science identity (competence/performance beliefs, science interest, and external recognition; Guo et al., 2022) represents person inputs, and (3) STEM career interest represents the motivational outcome.

Informed by this theoretical rationale and empirical precedent, the study formulates the following hypotheses:

**H1.**  *School belonging in a science-oriented educational environment positively predicts students’ interest in STEM careers.*

**H2.**  *Science identity mediates the relationship between school belonging and STEM career interest.*

**H3.**  *Competence/performance beliefs significantly mediate the relationship between school belonging and STEM career interest.*

**H4.**  *Science interest significantly mediates the relationship between school belonging and STEM career interest.*

**H5.**  *External recognition significantly mediates the relationship between school belonging and STEM career interest.*

By explicitly mapping SCCT constructs onto empirical variables and modeling science identity development as a key mediational process, this study aims to clarify whether school belongingness has an impact on STEM career interests, and also explores the manner in which this impact occurs—namely, the direct effect and the indirect effect through science identity. In doing so, it offers culturally grounded insights for educators and policymakers aiming to support adolescents’ long-term engagement in science-related fields.

## 2. Materials and Methods

### 2.1. Study Context and Sample

The present study was situated in a STEM-focused public high school in eastern China, officially designated as one of “X Province’s Model Schools for Science Education.” Recognized for its leadership in integrating formal and informal STEM learning, the school provides a rich educational context aligned with the theoretical concerns of this study. Within the framework of Social Cognitive Career Theory (SCCT) (Lent et al., 1994, 2003), this school exemplifies a supportive learning environment—a key contextual affordance—where students are afforded both institutional structure and personalized opportunities to cultivate their science identity and career aspirations.

A growing body of research highlights that the most effective STEM learning outcomes emerge when schools combine structured academic instruction with flexible, emotionally engaging informal activities (Adams & Gupta, 2017; Balyk et al., 2022; Porter et al., 2024). The focal school reflects this integrated approach. Formal components include advanced science courses, elective “doctoral classes” taught by PhD or postdoctoral researchers, and project-based inquiry modules embedded into the curriculum. Informal opportunities include student-led science clubs, immersive summer camps, interdisciplinary design challenges, and intensive coaching for national science competitions. These activities foster authentic engagement with scientific practices and social communities—conditions conducive to the development of competence beliefs, science-related interest, and recognition as a “science person” (Carlone & Johnson, 2007; Guo et al., 2022).

A total of 451 students participated in this study (see Table 1 for demographic details). All students had prior experience with at least one school-sponsored STEM activity, ensuring relevance to the constructs under investigation. While the school attracts academically high-achieving students from across the province, the sample includes a range of grade levels (grades 10–12), genders, and science orientations. This diversity allows for meaningful examination of how individual variation in school experiences contributes to the formation of science identity and STEM career interest.

To validate the STEM-rich environment of the school, a supplementary survey was conducted with a representative subset of 110 students randomly drawn from the main sample of 451. This design ensured manageable survey administration while maintaining representativeness.

Participation in the study was voluntary. Informed consent was obtained from all students and their guardians. Participant anonymity was protected throughout the process, and all procedures were reviewed and approved by the Ethics Committee of Beijing Normal University (Approval ID: BNU202308100026, dated 25 August 2023), in accordance with the Declaration of Helsinki.

The final sample included 451 students. The gender distribution was 60.8% male (*n* = 275) and 39.2% female (*n* = 176), which likely reflects higher participation rates of male students in STEM-related extracurricular activities within this context. Regarding grade levels, 74.9% were in Grade 10 (*n* = 338) and 25.1% in Grade 11 (*n* = 113). Grade 12 students were not included due to the timing of data collection, which coincided with their intensive preparation for the national college entrance examination (Gaokao). This distribution also aligns with the structure of the Chinese high school curriculum, where students complete general science courses in Grade 10 before specializing in science-focused tracks in Grade 11. These demographic characteristics are summarized in Table 1.

### 2.2. Measures

The survey instruments used in this study were adapted from established, validated questionnaires. All original instruments were in English and were translated into Chinese to ensure comprehension by the participants. The translations were cross-checked by two PhD-level psychologists to ensure linguistic and conceptual accuracy.

#### 2.2.1. School Belonging

The Belong to School questionnaire compiled by Seyranian et al. (2018) was selected, which consists of 4 items, such as “I think this school is very suitable for me”. A 7-point Likert scale (1–7 points from “strongly disagree” to “strongly agree”) was used to calculate the average score, with higher scores indicating an individual’s higher sense of belonging to the school.

#### 2.2.2. STEM Career Interest

The STEM Career Interest Survey (STEM-CIS), particularly its science section developed by Kier et al. (2014), was utilized to measure students’ interest in pursuing STEM careers. The scale comprises 11 items. Responses were rated on a 5-point Likert scale (1 = “strongly disagree” to 5 = “strongly agree”), with higher scores reflecting greater interest in STEM careers. Example items include:

- “I plan to use science in my future career.”
- “I am interested in careers that use science.”

#### 2.2.3. Science Identity

Science identity was measured based on a multidimensional framework incorporating competence/performance beliefs, science interest, and external recognition. The Student Science Identity Questionnaire (SSI), developed by Chen and Wei (2022) and adapted by Guo et al. (2022), was used for this purpose. The SSI contains a series of items rated on a 5-point Likert scale (1 = “strongly disagree” to 5 = “strongly agree”). For analytical simplicity, scores across the three dimensions were summed and averaged, creating a composite indicator of science identity. This transformation reflects a first-order variable approach, with three exogenous observed variables representing the sub-dimensions of competence/performance beliefs, external recognition, and science interest. Example items include:

- Competence/performance beliefs: “I think I am good at science.”
- Science Interest: “I like to attend classes that are related to science.”
- External Recognition: “My family and friends recognize me as a science person.”

#### 2.2.4. Validation of the School’s STEM Orientation

To empirically validate the school’s designation as a STEM-focused institution, a supplementary survey was conducted with a subset of 110 students randomly drawn from the larger sample. Students rated their experiences with key STEM initiatives—the Science and Technology Association (STA), the Scientific Inquiry Project (SIP), and various Discipline-specific Competitions (DC)—as well as the quality of related teacher support. Items were scored on a 5-point Likert scale (1 = very poor/low, 5 = very good/high). Items were scored on a 5-point Likert scale (1 = very poor/low, 5 = very good/high). Example items include:

- “I actively participate in activities organized by the Science and Technology Association.”
- “My teachers provide useful guidance when I prepare for science competitions.”

### 2.3. Data Analysis Strategy

Data were managed and analyzed using SPSSAU software (version 25). The analytical process was conducted in three stages:

Data Management and Cleaning. Preliminary data entry and cleaning were performed to ensure accuracy and prepare for subsequent analyses.

Confirmatory Factor Analysis (CFA). CFA was conducted to examine the measurement properties of the latent constructs. Factor loadings, composite reliability (CR), and average variance extracted (AVE) were evaluated to assess convergent validity, while discriminant validity was examined by comparing the square root of AVE with inter-construct correlations.

Mediation Analysis. In line with our hypotheses, we tested a mediation model in which school belonging (independent variable) predicts STEM career interest (dependent variable), mediated by the three dimensions of science identity (competence/performance beliefs, science interest, and recognition). We employed bootstrapping with 5000 resamples to estimate indirect effects and their 95% confidence intervals. This approach provides a robust test of mediation effects without assuming normality of the indirect paths.

Monte Carlo power analysis. To further examine the robustness of the focal path effects, we conducted a Monte Carlo power analysis (Thoemmes et al., 2010). Using the standardized path coefficients and R^2^ values estimated from the empirical models as population parameters, we generated 2000 simulated datasets with the observed sample size (N = 451) at α = 0.05. For each dataset, the mediation model was re-estimated, and the proportion of significant results (*p* < 0.05) was taken as the empirical power.

## 3. Results

### 3.1. Measurement Model Results

#### 3.1.1. Reliability and Validity Testing

To assess the measurement quality of the constructs (school belonging, science identity, and STEM career interest), reliability and validity analyses were conducted. The internal consistency of each dimension was analyzed by Cronbach coefficient reliability test method (Li & Xin, 2008). As shown in Table 2, all values exceeded the recommended threshold of 0.70, indicating good reliability. Construct validity was further assessed by the Kaiser–Meyer–Olkin (KMO) measure and Bartlett’s test of sphericity. When KMO > 0.60 and Bartlett’s test is significant (*p* < 0.001), the data are considered suitable for factor analysis (Li & Xin, 2008). Results confirmed that the scales used in this study demonstrated good construct validity.

Multicollinearity diagnostics were also conducted to ensure that independent variables were not excessively correlated. Multicollinearity occurs when two or more independent variables exhibit substantial linear relationships, thus failing to provide unique explanatory power in regression models. The Variance Inflation Factor (VIF) and tolerance values are commonly used to detect this issue, with VIF > 5 or tolerance < 0.2 typically indicating problematic multicollinearity. In this study, all VIF values were below 5 and tolerance values above 0.2, suggesting no serious multicollinearity among the independent variables.

#### 3.1.2. Convergent and Discriminant Validity

Convergence validity analysis can use AVE and CR. If the AVE value of each Factor is greater than 0.5, and the CR value is greater than 0.7, it indicates that it has good convergence validity. Differential validity emphasizes that measures that should not be in the same factor are indeed not under the same factor. Discriminant validity was assessed using the Fornell–Larcker criterion. When the AVE square root value of each factor was greater than “the maximum value of the correlation coefficient between the factor and other factors”, it indicated that it had good discriminative validity. The results show that the three latent variables all met the recommended standards (Luo & Jiang, 2014), as shown in Table 3.

#### 3.1.3. Confirmatory Factor Analysis (CFA)

A confirmatory factor analysis (CFA) was performed to test the hypothesized three-factor measurement model (school belonging, science identity, and STEM career interest, see Table 4). The results showed good model fit: CMIN/df = 2.958, RMSEA = 0.066, RMR = 0.038, AGFI = 0.903, and CFI = 0.963, meeting commonly accepted thresholds (Hou et al., 2004). These findings indicate that the three constructs are empirically distinguishable and adequately measured by their observed indicators.

Taken together, the results of convergent validity, discriminant validity, and CFA fit indices provide strong evidence that the three latent constructs are conceptually and empirically distinct within the present school context.

### 3.2. Validation of the STEM Learning Environment

#### 3.2.1. Quantitative Indicators

**Figure 1:**
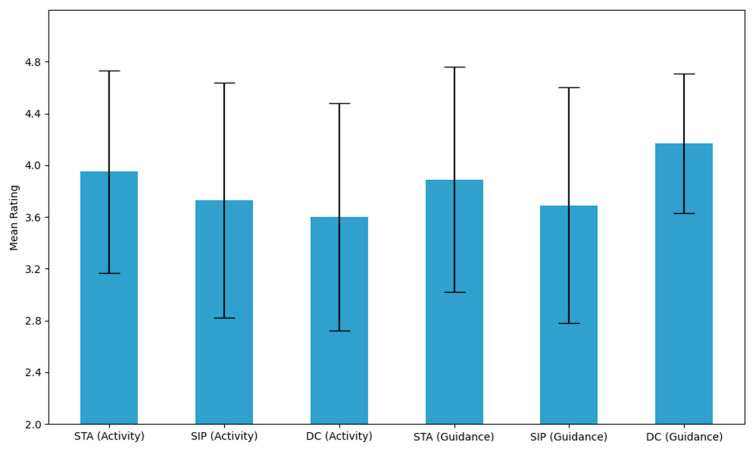
Student Perceptions of STEM Activities and Teacher Guidance (error bars represent SDs).

As described in Section 2.2.4, student perceptions of the school’s STEM activities (STA, SIP, DC) and related teacher guidance were assessed on a 5-point Likert scale. Results from the 110-student subset are summarized in Table 5 and Figure 1. On average, students reported favorable perceptions of both the activities themselves (M = 3.76) and the related teacher support (M = 3.92).

Among the STEM activities, the Science and Technology Association (STA) received the highest rating (M = 3.95, SD = 0.78), followed by the Scientific Inquiry Project (SIP) (M = 3.73, SD = 0.91), and Discipline Competitions (DC) (M = 3.60, SD = 0.88). The greater standard deviation for SIP suggests higher variability in individual experience, possibly due to differences in project scope or execution.

Teacher guidance was evaluated even more positively. Notably, support for Discipline Competitions received the highest rating of all items (M = 4.17, SD = 0.54), indicating both high quality and consistency. Guidance for STA (M = 3.89, SD = 0.87) and SIP (M = 3.69, SD = 0.91) also received favorable evaluations.

#### 3.2.2. Interpretation and Contextual Relevance

These findings confirm that the focal school is perceived by students as offering a robust STEM learning environment. Both programmatic opportunities (e.g., clubs, inquiry projects, competitions) and pedagogical support (e.g., teacher mentoring and coaching) were evaluated favorably, reinforcing the school’s reputation as a leading STEM institution. The especially high ratings for teacher guidance in competitive activities suggest that active mentorship is a central cultural feature of this school, likely fostering students’ confidence, motivation, and recognition as “science people.” This positive evaluation also establishes a strong contextual foundation for the present study, strengthening the rationale for investigating how school belonging and science identity operate within such an affirmed STEM-oriented ecosystem to shape students’ career interests.

### 3.3. Mediation Analysis: Direct and Indirect Effects

Before testing the hypothesized mediation pathways, confirmatory factor analysis and tests of convergent and discriminant validity (see Section 3.1) confirmed that school belonging, science identity, and STEM career interest are conceptually and empirically distinct constructs. This provides a robust basis for the mediation analysis. Table 6 presents the results of the direct and mediating effect analyses.

**Figure 2:**
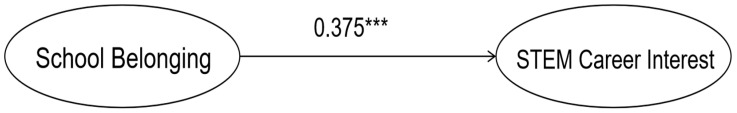
Coefficient of direct effect (Model 1). Note: *** *p* < 0.001.

Direct effect (Model 1, see Figure 2). To evaluate H1, we first tested the direct effect of school belonging on STEM career interest without including science identity. Results showed that school belonging significantly predicted STEM career interest (β = 0.375, t = 8.576, *p* < 0.001; R^2^ = 0.139), thus supporting H1.

**Figure 3:**
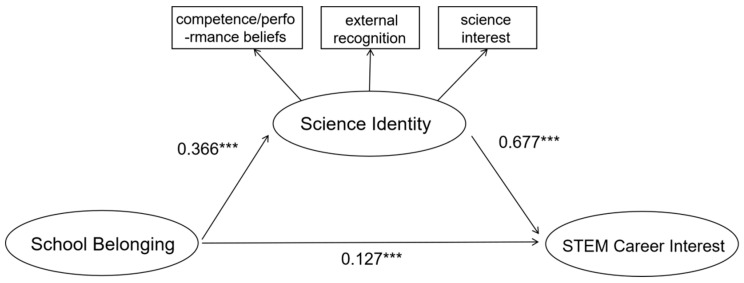
Coefficient of mediating effect (Model 2). Note: *** *p* < 0.001.

Overall mediation model (Model 2, see Figure 3). Next, we examined whether science identity mediated this relationship (H2). When science identity was included, the direct effect of school belonging on STEM career interest remained significant but was substantially reduced (β = 0.127, t = 3.680, *p* < 0.001). Meanwhile, school belonging positively predicted science identity (β = 0.366, t = 8.342, *p* < 0.001), and science identity strongly predicted STEM career interest (β = 0.677, t = 19.630, *p* < 0.001). A bootstrap analysis with 5000 resamples (Wen & Ye, 2014) confirmed the mediating role of science identity, with a bias-corrected 95% confidence interval for the indirect effect of [0.114, 0.210], which did not include zero. The standardized direct and indirect effects accounted for 33.85% and 66.15% of the total effect, respectively, indicating that science identity serves as a substantial partial mediator in this process, thus supporting H2. To further establish the robustness of these findings, a Monte Carlo power analysis (2000 replications, N = 451, α = 0.05) demonstrated that the focal path effects were well-powered (all > 0.95), suggesting that the observed effects represent true associations rather than chance results (Muthén & Muthén, 2002; Thoemmes et al., 2010).

Individual mediation models (Models 3–5). To further examine the specific role of each component of science identity, we tested competence/performance beliefs, external recognition, and science interest as mediators in separate models (H3–H5). In each case, school belonging significantly predicted the identity dimension, which in turn significantly predicted STEM career interest. Bootstrap analyses confirmed that all three indirect effects were statistically significant: the 95% confidence intervals were [0.1412, 0.2722] for competence/performance beliefs, [0.0554, 0.1500] for external recognition, and [0.1923, 0.3432] for science interest. Because none of these intervals contained zero, the results indicate that each dimension of science identity partially mediated the association between school belonging and STEM career interest. These findings provide support for H3, H4, and H5.

**Figure 4:**
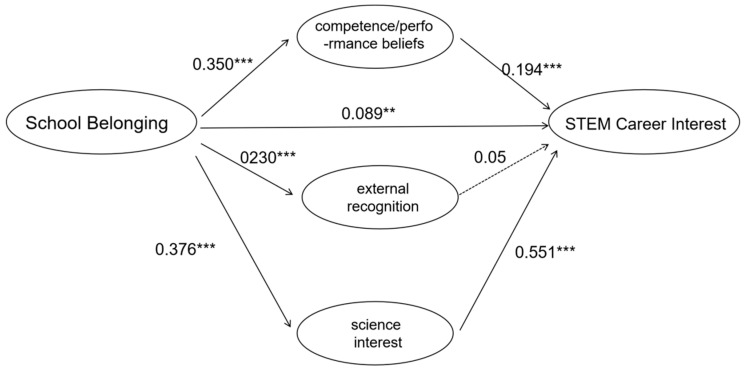
Coefficient of parallel mediating effect (Model 6). Note: ** *p* < 0.01, *** *p* < 0.001.

Parallel mediation model (Model 6, see Figure 4). When all three dimensions of science identity were entered simultaneously, their relative contributions became clearer. Science interest (β = 0.551, t = 12.478, *p* < 0.001) and competence/performance beliefs (β = 0.194, t = 4.088, *p* < 0.001) remained significant predictors of STEM career interest, whereas external recognition did not (β = 0.050, t = 1.250, *p* > 0.05). Bootstrap confidence intervals supported these findings. The indirect effects through competence/performance beliefs [0.1008, 0.2874] and science interest [0.4642, 0.6377] were significant, while the interval for external recognition [–0.0286, 0.1292] included zero. This indicates that external recognition did not serve as a significant mediator when considered alongside the other two dimensions.

**Figure 5:**
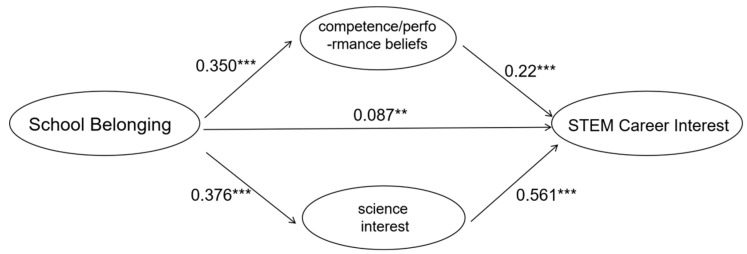
Coefficient of parallel mediating effect (Model 7). Note: ** *p* < 0.01, *** *p* < 0.001.

Final model (Model 7, see Figure 5). To further clarify these dynamics, we specified a model excluding external recognition and compared the relative contributions of competence/performance beliefs and science interest. Results showed that the direct effect of school belonging on STEM career interest was small but significant (β = 0.0873, t = 2.669, *p* < 0.01). Importantly, the indirect effect through science interest (0.2110) was markedly larger than that through competence/performance beliefs (0.0769).

Together, these results underscore that science interest is the dominant pathway linking school belonging to STEM career interest, while competence/performance beliefs play a secondary role and external recognition exerts limited influence in this context.

## 4. Discussion

This study drew on data from 451 Chinese high school students to develop a mediating model that examined how school belonging influences students’ interest in STEM careers, with science identity as a mediating factor. The findings contribute to a deeper understanding of the psychological and sociocultural mechanisms that shape adolescents’ STEM engagement.

### 4.1. Dual Pathways: Direct and Indirect Effects of School Belonging

The results revealed two distinct pathways through which school belonging promotes STEM career interest: a direct effect and an indirect effect mediated by science identity. Students who feel accepted and supported at school are more likely to participate in science-related activities, maintain interest, and envision future careers in STEM.

In line with Social Cognitive Career Theory (SCCT) (Lent et al., 2003), school belonging functions as a contextual affordance that generates formative learning experiences. Supportive school climates provide opportunities for mastery, credible encouragement, vicarious role models, and reduced anxiety—conditions that strengthen self-efficacy (belief in one’s capability to succeed in science) and foster positive outcome expectations (belief that engaging in science will lead to valued rewards). These psychological resources are then internalized within students’ science identity, which channels current experiences into long-term STEM aspirations. In this way, belonging operates not simply as an emotional state but as an enabling context that shapes the motivational pathways emphasized in SCCT.

This process was especially evident in the current study’s setting—a science-focused high school where structured mentorship, collaborative research, and a student-centered culture collectively foster belonging and motivation. These findings demonstrate that, despite critiques of exam-driven schooling, well-designed institutional environments can transform belonging into sustained motivation and career vision. In the Chinese context, this resonates with recent reforms such as the 2022 Science Curriculum Standards, which emphasize inquiry-based, interdisciplinary, and value-oriented science education. Such policies advocate learning environments that integrate achievement with belonging, identity, and long-term interest in science (Cheng, 2014; Fu, 2014; Jin, 2012; Parr et al., 2020). While the present section focuses on the micro-level mechanisms of belonging, their broader embedding in China’s cultural and policy environment will be elaborated in Section 4.3.

### 4.2. Science Identity as a Mediator: Unequal Contributions of Its Components

The mediation analysis revealed that the three components of science identity contributed unevenly to STEM career interest: science interest emerged as the strongest mediator, followed by competence/performance beliefs, while external recognition had the weakest influence. These findings offer important insights into the mechanisms of identity-based motivation in the Chinese context.

#### 4.2.1. Science Interest as the Core Motivator

The dominant role of science interest can be understood through the integration of three complementary perspectives. First, Self-Determination Theory (SDT) (Deci & Ryan, 2000) highlights that interest grounded in autonomy, competence, and relatedness supports intrinsic motivation. Second, Social Cognitive Career Theory (SCCT) posits that contextual affordances shape self-efficacy and outcome expectations, which in turn sustain career interests (Lent et al., 1994, 2016). Third, interest development theory (Hidi & Renninger, 2006) emphasizes that sustained interest emerges when learners repeatedly engage with meaningful content that reinforces their sense of competence and control.

In China, these psychological processes are embedded within a mastery-oriented educational culture and increasingly supported by structural opportunities for inquiry. High-stakes examinations and collective honor norms strongly incentivize students to pursue mastery in science, while recent reforms (e.g., the 2022 Science Curriculum Standards and the STEM Education 2035 Action Plan) expand opportunities for authentic engagement through hands-on projects, competitions, and mentorship. In this ecology, interest does not operate in isolation: it co-evolves with students’ outcome expectations—for example, perceived pathways to research participation, competition-based advancement, recommendation letters, and portfolio-building. This coupling of interest with tangible academic and career opportunities helps explain why science interest emerged as the most potent mediator of career intentions in our model.

Together, these converging mechanisms illustrate how science interest functions as the primary psychological driver of STEM career motivation in high-resource Chinese contexts, where recognition is abundant and competence is heavily validated through examinations.

#### 4.2.2. External Recognition: Contextually Limited

The comparatively weak role of external recognition contrasts with prior studies where recognition was a strong predictor of science identity and aspirations (Hazari et al., 2010). In the present study, this attenuation likely reflects context-specific measurement and sociocultural norms.

First, our measurement scope was narrow, emphasizing proximal recognition from teachers, parents, and peers. This left out broader forms of institutional and cultural recognition—such as school-wide awards, symbolic rituals, media portrayals, and policy discourse—which may carry significant weight in shaping identity through status signals.

Second, in an elite, high-achieving school, recognition is pervasive and routinized. Awards, praise, and public affirmation are frequent, creating a normalization or ceiling effect that diminishes their marginal psychological salience. In such ecologies, students’ motivation is increasingly anchored in mastery and intrinsic interest rather than external praise.

Third, recognition may sometimes be conditional, tied to strict performance thresholds or competitive benchmarks. For some students, this produces pressure-induced belonging—a sense of acceptance contingent on achievement—which can transform recognition into performance stress, thereby weakening its motivational yield. This suggests a potential avenue for future research on the interplay between conditional belonging and recognition.

Importantly, these findings do not contradict prior frameworks but rather indicate contextual reweighting. In recognition-saturated settings, interest and competence/performance beliefs may absorb some of recognition’s motivational functions. Conversely, in low-resource or marginalized populations, external recognition may play a far more decisive role in affirming identity and sustaining motivation. This underscores that the relative salience of identity components is ecologically contingent—a reflection of cultural and institutional conditions rather than a universal pattern.

### 4.3. Cultural and Policy Contexts: Structural Enablers

The observed effects in this study cannot be understood apart from the distinctive features of China’s educational culture. In the Chinese educational context, school belonging and science identity are shaped not only by broad cultural values but also by concrete institutional practices that may help explain the patterns observed in our findings.

One relevant factor may be the exam-oriented culture, in which academic performance is central to school life and belonging is often tied to collective achievement in high-stakes examinations. Such a system may reinforce competence/performance beliefs and align identity formation with measurable markers of success.

Another consideration is the prevalence of institutionalized recognition practices, such as awards, rankings, public commendations, and collective honors. While these practices make recognition highly visible, their routinization may reduce marginal psychological salience. This dynamic may help explain why external recognition played a weaker role in our model compared with science interest and competence beliefs.

The class collective system and teacher authority structures also provide important context. Homeroom teachers, serving simultaneously as academic leaders and moral exemplars, often embed belonging within shared routines, group honor systems, and moral guidance. These features may shape the kinds of mastery experiences, vicarious learning, and social persuasion that SCCT identifies as drivers of self-efficacy and outcome expectations.

Finally, the national strategic orientation toward science and innovation amplifies these school-level dynamics. Policies such as the 2022 Science Curriculum Standards and the STEM Education 2035 Action Plan have encouraged inquiry-based learning, project participation, and innovation competitions. For students, these policy-driven opportunities may couple science interest with outcome expectations—for example, through competition-based advancement, research opportunities, or innovation portfolios—helping to transform intrinsic curiosity into sustained STEM career aspirations.

Taken together, these features of China’s educational system suggest that school belonging and science identity are not abstract psychological states but are deeply shaped by cultural logics and institutional practices. Our findings therefore extend SCCT by showing how contextual affordances—filtered through exam culture, recognition regimes, class collectives, and national strategies—may reweight the salience of identity components. This helps explain why science interest and competence beliefs emerged as stronger mediators, while recognition played a comparatively weaker role in this particular setting.

## 5. Implications

This study highlights that school belonging not only directly predicts STEM career interest but also operates indirectly through science identity, with science interest emerging as the strongest mediator and external recognition playing a limited role. These findings suggest several context-specific implications for strengthening STEM education.

### 5.1. Embedding Belonging in Classroom and School Culture

The results highlight that school belonging is not merely an abstract feeling but becomes motivational when translated into everyday learning practices. In concrete terms, classrooms should provide structured opportunities for students to experience competence—for example, scaffolded experiments where success is attainable at multiple stages rather than only at the final outcome. Belonging also grows when students encounter relatable role models: alumni talks, near-peer mentoring, or collaboration with older students can make “being a science person” visible and attainable. Finally, teacher feedback in exam-oriented settings often focuses on errors or scores; our findings suggest that feedback framed as encouragement and recognition of effort is especially powerful in reinforcing self-efficacy and sustaining motivation.

### 5.2. Fostering Science Interest Through Sustained Inquiry Opportunities

The finding that science interest emerged as the strongest mediator highlights the importance of cultivating students’ curiosity and enjoyment of science through sustained, practice-based opportunities. Rather than relying on one-off activities or high-stakes competitions, schools can embed interest development into everyday learning experiences. For example, classroom projects can be extended into school-wide science fairs, exhibitions, or community presentations, providing students with authentic opportunities to share their work and feel that their curiosity matters beyond test performance. Regular laboratory sessions, maker activities, and problem-solving workshops can offer repeated occasions for exploration and mastery, helping students translate curiosity into competence.

Teachers can also encourage students to maintain project portfolios or science journals, where they record progress, reflect on challenges, and connect their learning to personal goals. These practices reinforce the sense that scientific interest is not episodic but accumulates into a durable self-perception of being capable in science. In addition, inviting local STEM professionals or university students to participate in classroom or extracurricular activities can make future pathways more visible and relatable. Such structural supports provide both mastery experiences and credible role models, thereby strengthening self-efficacy and outcome expectations within the SCCT framework.

By normalizing inquiry, reflection, and recognition within ordinary school routines, these practices help ensure that science interest functions as a stable motivational force that connects present learning to long-term STEM aspirations.

### 5.3. Rethinking Recognition Beyond Awards

Our finding that external recognition had limited influence does not mean it is unimportant, but that in high-achieving schools, its value may be diminished by over-saturation. Recognition in such environments often takes the form of frequent awards, rankings, and public praise—what we might call “normalized recognition.” This can create a ceiling effect where additional recognition carries little new meaning. In some cases, recognition may even feel conditional, tied to strict performance thresholds, and thus translate into pressure rather than support. A more productive approach would be to diversify what is recognized—for example, highlighting persistence in experiments, teamwork in projects, or creativity in problem-solving. While recognition may be less salient in elite settings, in under-resourced schools or for marginalized groups, it could remain a critical driver, suggesting that its effectiveness is highly context-dependent

## 6. Limitations

Several limitations should be noted. First, the study was conducted in a single, selective high school with strong STEM resources, which may have amplified the observed effects and limited the generalizability of the findings. Second, the cross-sectional design precludes causal inference, making it difficult to rule out reverse causality between school belonging and STEM career interest. Finally, all measures relied on student self-reports, raising the possibility of social desirability bias and common method variance. Although validated instruments and anonymous responses were used to mitigate these risks, they cannot be fully eliminated. Future research should address these issues by examining more diverse school contexts, employing longitudinal or experimental designs, and incorporating multiple data sources.

## 7. Conclusions

This study examined how school belonging influences Chinese high school students’ STEM career interest and the mediating role of science identity within this process. Drawing on Social Cognitive Career Theory (SCCT), we operationalized contextual affordances as school belonging, person inputs as science identity, and career interest as students’ STEM aspirations. The findings highlight that school belonging not only exerts a direct effect on STEM career interest but also an indirect effect through science identity, with science interest emerging as the strongest mediator. These results underscore the importance of supportive school environments in fostering students’ competence beliefs, intrinsic interest, and recognition as “science people.” By focusing on a STEM-oriented high school in China, this study extends existing literature on school belonging and science identity into a non-Western context, thereby offering a culturally grounded perspective on adolescent STEM engagement. The findings contribute theoretically by clarifying the mechanisms through which psychosocial experiences are translated into long-term career aspirations, and practically by pointing to the value of cultivating inclusive, identity-affirming school climates. Taken together, the study provides empirical support for the argument that fostering school belonging and strengthening science identity are critical levers for sustaining students’ STEM career interest, with implications for both educational practice and talent development policy.

## Figures and Tables

**Table 1:** Participant Demographics (N = 451).

Category	Basic Count	Actual Count	Percentage (%)
Gender			
Male	275	275	60.98
Female	176	176	39.02
Grade			
Grade 10	338	338	74.94
Grade 11	113	113	25.06

**Table 2:** Results of Reliability, Validity and Multicollinearity Tests.

Variables	Cronbach’s Alpha	KMO	Bartlett	VIF	Tolerance
School Belonging	0.89	0.82	1098.046 ***	1.185	0.844
STEM Career Interest	0.90	0.88	1730.814 ***	2.362	0.423
Science Identity	0.94	0.90	4392.075 ***	2.474	0.404

Note: *** *p* < 0.001.

**Table 3:** Convergent and Discriminant Validity Analysis.

Variables	CR	AVE	Factor Loading	Correlation Coefficient and AVE Square Root		
				School Belonging	STEM Career Interest	Science Identity
School Belonging	0.90	0.68	0.752~0.904	(0.83)		
STEM Career Interest	0.95	0.62	0.744~0.881	0.36 **	(0.78)	
Science Identity	0.97	0.59	0.784~0.975	0.39 **	0.76 **	(0.77)

Note: The number in parentheses is the square root of the latent variable AVE; ** *p* < 0.01.

**Table 4:** Confirmatory Factor Analysis (CFA) Model fit indices.

CMIN	DF	*p* Value	CMIN/df	RMSEA	RMR	AGFI	CFI
440.722	149.000	0.000	2.958	0.066	0.038	0.903	0.963

Note: CMIN/df < 3 is satisfactory, 3–5 is acceptable; RMSEA < 0.05 is satisfactory, and 0.05~0.08 is acceptable. RMR < 0.05 satisfactory, 0.05~0.08 acceptable; AGFI > 0.90 satisfactory, 0.80~0.90 acceptable; CFI > 0.90 satisfactory, 0.80~0.90 acceptable.

**Table 5:** Student Perceptions of STEM Activities and Teacher Guidance (N = 110).

Category	Item	Description	Mean	SD
STEM Activities	Total (STEM Activities)		3.76	—
	Science and Technology Association (STA)	Participation in robotics clubs, coding groups, science fairs, and tech talks organized by the STA.	3.95	0.78
	Scientific Inquiry Project (SIP)	Engagement in semester-long research projects, experiment design, data analysis, and writing research reports.	3.73	0.91
	Discipline Competition (DC)	Experience in subject-specific contests like Math Olympiads, Physics Bowls, and Chemistry tournaments.	3.60	0.88
Teacher Guidance	Total (Teacher Guidance)		3.92	—
	Teachers’ guidance (for STA)	Support in club management, project advising, science fair preparation, and technical assistance.	3.89	0.87
	Teachers’ guidance (for SIP)	Mentorship in research design, experimental procedures, data interpretation, and report feedback.	3.69	0.91
	Teachers’ guidance (for DC)	Coaching for competitions, strategy training, mock exams, and individualized guidance.	4.17	0.54
	Overall Mean		3.84	—

Note. Data are based on a representative subset of 110 students randomly drawn from the full sample of 451 to validate the STEM orientation of the school.

**Table 6:** Direct and Mediating Effect Analysis.

Model	Consequent Variable	Predictor Variable	R^2^	F	β	t
1	STEM Career Interest	School Belonging	0.139	73.543	0.375	8.576 ***
2	STEM Career Interest	School Belonging	0.536	260.925	0.127	3.680 ***
	STEM Career Interest	Science identity			0.677	19.630 ***
	Science identity	School Belonging	0.132	69.595	0.366	8.342 ***
3	STEM Career Interest	School Belonging	0.442	179.124	0.169	4.503 ***
	STEM Career Interest	competence/performance beliefs			0.588	15.645 ***
	competence/performance beliefs	School Belonging	0.121	62.676	0.350	7.917 ***
4	STEM Career Interest	School Belonging	0.325	107.881	0.274	6.860 ***
	STEM Career Interest	external recognition			0.441	11.061 ***
	external recognition	School Belonging	0.51	25.120	0.230	5.012 ***
5	STEM Career Interest	School Belonging	0.570	299.573	0.109	3.263 **
	STEM Career Interest	science interest			0.709	21.255 ***
	science interest	School Belonging	0.139	73.824	0.376	8.592 ***
6	STEM Career Interest	School Belonging	0.594	165.366	0.089	2.709 **
	STEM Career Interest	competence/performance beliefs			0.194	4.088 ***
	STEM Career Interest	external recognition			0.05	1.25
	STEM Career Interest	science interest			0.551	12.478 ***
	competence/performance beliefs	School Belonging	0.121	62.676	0.350	7.917 ***
	external recognition	School Belonging	0.51	25.120	0.230	5.012 ***
	science interest	School Belonging	0.139	73.824	0.376	8.592 ***
7	STEM Career Interest	School Belonging	0.593	219.683	0.087	2.669 **
	STEM Career Interest	competence/performance beliefs			0.22	5.118 ***
	STEM Career Interest	science interest			0.561	12.946 ***
	competence/performance beliefs	School Belonging	0.121	62.676	0.350	7.917 ***
	science interest	School Belonging	0.139	73.824	0.376	8.592 ***

Note: *** *p* < 0.001; ** *p* < 0.01.

## Data Availability

The data are not publicly available due to privacy concerns. The datasets generated and analyzed during the current study are available from the corresponding author on reasonable request.
